# Prion protein polymorphisms in Michigan white-tailed deer (*Odocoileus virginianus*)

**DOI:** 10.1080/19336896.2021.1990628

**Published:** 2021-11-09

**Authors:** Caitlin N. Ott-Conn, Julie A. Blanchong, Wes A. Larson

**Affiliations:** a Michigan Department of Natural Resources, Wildlife Disease Laboratory, Lansing, USA; b Department of Natural Resource Ecology and Management, Iowa State University, Ames, IA, USA; c National Oceanographic and Atmospheric Administration, National Marine Fisheries Service, Alaska Fisheries Science Center, Juneau, AK, USA

**Keywords:** Cervid, chronic-wasting disease, Michigan, prion, *Odocoileus virginianus*, prion protein gene, PRNP, transmissible spongiform encephalopathy, white-tailed deer

## Abstract

Chronic Wasting Disease (CWD), a well-described transmissible spongiform encephalopathy of the *Cervidae* family, is associated with the aggregation of an abnormal isoform (PrP^CWD^) of the naturally occurring host prion protein (PrP^C^). Variations in the PrP gene (*PRNP*) have been associated with CWD rate of infection and disease progression. We analysed 568 free-ranging white-tailed deer (*Odocoileus virginianus*) from 9 CWD-positive Michigan counties for *PRNP* polymorphisms. Sampling included 185 CWD-positive, 332 CWD non-detected, and an additional 51 CWD non-detected paired to CWD-positives by sex, age, and harvest location. We found 12 polymorphic sites of which 5 were non-synonymous and resulted in a change in amino acid composition. Thirteen haplotypes were predicted, of which 11 have previously been described. Using logistic regression, consistent with other studies, we found haplotypes C (OR = 0.488, 95% CI = 0.321–0.730, P < 0.001) and F (OR = 0.122, 95% CI = 0.007–0.612, P < 0.05) and diplotype BC (OR = 0.340, 95% CI = 0.154–0.709, P < 0.01) were less likely to be found in deer infected with CWD. As has also been documented in other studies, the presence of a serine at amino acid 96 was less likely to be found in deer infected with CWD (P < 0.001, OR = 0.360 and 95% CI = 0.227–0.556). Identification of *PRNP* polymorphisms associated with reduced vulnerability to CWD in Michigan deer and their spatial distribution can help managers design surveillance programmesand identify and prioritize areas for CWD management.

## Introduction

Chronic Wasting Disease (CWD), a well described, fatal, transmissible spongiform encephalopathy of the *Cervidae* family, is associated with the aggregation of an abnormal isoform (PrP^CWD^) of the naturally occurring host prion protein (PrP^C^) [1–3]. First characterized in 1980 based on clinical and pathological findings in Colorado captive mule deer [2], CWD has since spread within the United States, been found in Canada and Europe, and been detected in imported cervids in Korea [4–7].

CWD is efficiently transmitted both horizontally [8–11] and vertically [12] with effective transmission between cervid species [6,13,14]. Prion shedding can begin during the pre-clinical stage of disease [9,15] through bodily fluids and excreta [9] and shed prions are able to be taken up by vegetation [16] and withstand degradation [17]. Once an animal is infected, CWD is always fatal [3].

CWD prevalence in free-ranging cervid populations has been found to be as high as 35% [18] with population-level impacts seen with prevalence as low as 13% [19–21]. Cervid populations provide not only social and cultural benefits through hunting and viewing, and ecological contributions to biodiversity, they also serve as a financial keystone species for conservation and management making their potential decline of considerable management concern.

Two non-synonymous polymorphisms within the prion gene (*PRNP*) resulting in changes to amino acids 95 (Q95H) and 96 (G96S), have been most commonly found to be associated with reduced disease susceptibility in white-tailed deer [22–35]. While neither have been shown to provide complete protection from CWD infection, they have been linked to reductions in genotype-specific prevalence rates [26, 29–31,36] or increased duration of incubation [37].

CWD was first detected in wild white-tailed deer (*Odocoileus virginianus*) in Michigan in 2015 through opportunistic passive surveillance, 6 years after the state’s first detection in a captive herd. Since 2015, Michigan has invested in intensive surveillance through localized culling and hunter assisted sampling and CWD has been detected in 9 counties at the time of this study. We examined the current frequency of *PRNP* polymorphisms among CWD-positive and non-detected deer in 9 CWD-positive Michigan counties, one county in the Upper Peninsula and 8 contiguous counties in central Michigan. We tested for an association between CWD status and *PRNP* polymorphisms and hypothesized CWD polymorphisms associated with reduced CWD infection are present in Michigan white-tailed deer.

## Results

**Figure 1.**
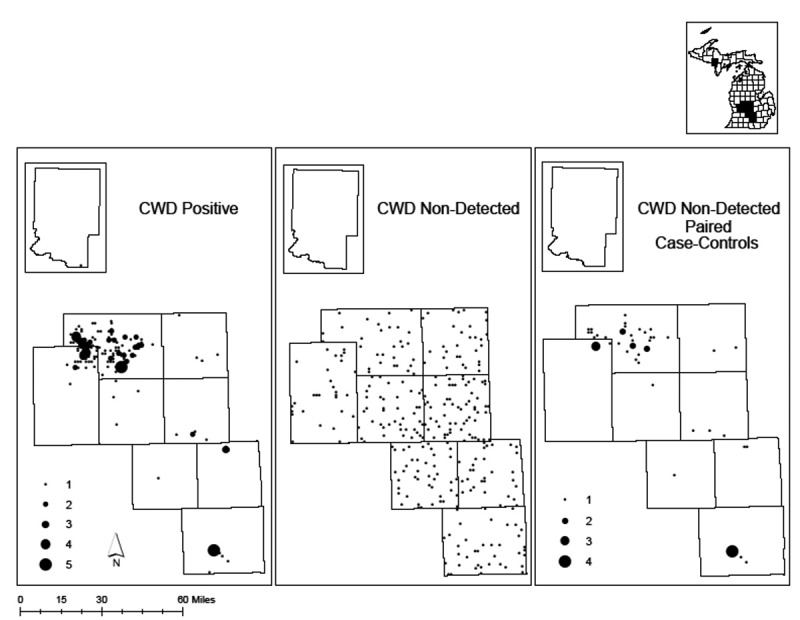
Distribution of sampled (a) chronic wasting disease (CWD) positive, (b) CWD non-detected, and (c) CWD non-detected paired control free-ranging white-tailed deer (*Odocoileus virginianus*) from 9 CWD-positive Michigan counties

*PRNP* sequences were determined for 568 free-ranging white-tailed deer from 9 CWD-positive Michigan counties. Of these samples, 185 were CWD-positive, 332 were CWD non-detected, and an additional 51 CWD non-detected were paired to CWD-positives to control for sex, age, and harvest location (Figure 1). Within the analysed 625bp region of the *PRNP* gene, we detected 12 single nucleotide polymorphisms (SNPs), 9 of which had been previously reported [22, 29, 33, 36, 38–41]. Of the 12 SNPs, 5 were non-synonymous, resulting in a change to the amino acid sequence (Table 1). BLAST and literature searches indicated that 589A/G, 642 G/A, and 643 C/A had not previously been reported. Full associated sequences have been deposited in GenBank under accession numbers MZ913400 – MZ913401.

**Table 1.** Thirteen haplotypes and associated single nucleotide polymorphisms (SNP) of the *PRNP* gene from 568 white-tailed deer (*Odocoileus virginianus*) from 9 CWD-positive Michigan counties. Bold text indicated non-synonymous SNPs. Asterisks indicate previously unreported SNPs

Haplotype	60	153	285	286	324	438	499	555	589*	642*	643*	676
A	C	C	A	G	A	C	A	C	A	G	C	C
B	C	C	A	G	A	C	A	T	A	G	C	C
C	C	C	A	A	A	C	A	T	A	G	C	C
D	C	T	A	G	A	C	A	C	A	G	C	C
E	C	C	A	G	A	T	A	C	A	G	C	C
F	T	C	C	G	A	C	A	C	A	G	C	C
G	T	C	A	G	A	C	A	C	A	G	C	C
J	C	C	A	G	G	C	A	C	A	G	C	C
K	T	C	A	G	A	C	A	C	A	G	C	A
MI-1	C	C	A	A	A	C	A	T	G	G	C	C
MI-2	C	T	A	G	A	C	A	C	A	A	A	C
O	T	T	A	G	A	C	A	C	A	G	C	C
OVC1	C	C	A	A	A	C	C	T	A	G	C	C
Non-synonymous Change	-	-	Q95H	G96S	-	-	-	-	K197E	-	Q215K	Q226K
Codon	-	-	95	96	-	-	-	-	197	-	215	226

Thirteen haplotypes were predicted from the 12 SNPs, 11 of which have previously been described [22, 40, 41]. Of the 13 haplotypes, B was most common (n = 368) and was used as the reference in logistic regression. Haplotypes J and MI-1 were found only in CWD non-detected deer, precluding them from analysis. Haplotypes C (OR = 0.488, 95% CI = 0.321–0.730, P < 0.001) and F (OR = 0.122 and 95% CI = 0.007–0.612, P < 0.05) were less likely to be found in deer infected with CWD (Table 2).

**Table 2.** *PRNP* haplotype frequency (f) and count for chronic wasting disease positive (+) and non-detected (-) white-tailed deer (*Odocoileus virginianus*) from 9 CWD-positive Michigan counties. Odds ratios and 95% confidence intervals are shown for significant variables (P < 0.05) determined by logistic regression against the most frequent haplotype, B. Asterisks indicate haplotypes found in only positive or non-detected deer precluding them from analysis. Bolding indicates previously unreported haplotypes

Haplotype	f	(+)	(-)	P-val	Odds Ratio
A	0.239	100	171	0.682	-
B	0.324	130	238	-	-
C	0.167	40	150	<.001	0.488 (0.321–0.730)
D	0.085	33	64	0.811	-
E	0.068	32	45	0.302	-
F	0.014	1	15	0.043	0.122 (0.007–0.612)
G	0.034	8	31	0.068	-
J*	0.003	0	3	-	-
K	0.020	12	11	0.109	-
MI-1*	0.001	0	1	-	-
**MI-2**	0.015	3	14	0.147	-
O	0.023	9	17	0.942	-
OVC1	0.007	2	6	0.549	-

We identified 49 diplotypes with AB being the most common (*n* = 89) and used this as the reference in logistic regression. Twenty-four diplotypes were found only in positive or non-detected deer, precluding them from analysis. Of the remaining 25 diplotypes, BC (OR = 0.340 and 95% CI = 0.154–0.709, P < 0.01) was less likely to be found in deer infected with CWD (Table 3).

**Table 3.** *PRNP* diplotype frequency (f) and count for chronic wasting disease positive (+) and non-detected (-) white-tailed deer (*Odocoileus virginianus*) from 9 CWD-positive Michigan counties. Odds ratios and 95% confidence intervals are shown for significant variables (P < 0.05) determined by logistic regression against the most frequent diplotype, AB. Asterisks indicate diplotypes found in only positive or non-detected deer precluding them from analysis

Diplotype	f	(+)	(-)	P-val	Odds Ratio
All Samples					
AB	0.157	36	53	-	-
BC	0.113	12	52	0.005	0.340 (0.154–0.709)
BB	0.095	20	34	0.685	-
AC	0.072	12	29	0.222	-
AA	0.058	15	18	0.619	-
BD	0.056	14	18	0.745	-
AD	0.051	9	20	0.366	-
BE	0.049	13	15	0.576	-
CC	0.030	6	11	0.691	-
BG	0.026	5	10	0.603	-
CD	0.025	2	12	0.077	-
AE	0.021	3	9	0.31	-
EC	0.019	2	9	0.168	-
BK	0.018	5	5	0.563	-
AG	0.016	3	6	0.679	-
AO	0.016	4	5	0.816	-
CG*	0.014	0	8	-	-
ED	0.014	5	3	0.239	-
BMI-2	0.012	1	6	0.202	-
CF*	0.012	0	7	-	-
BO	0.012	1	6	0.202	-
DD	0.011	1	5	0.273	-
AMI-2	0.009	2	3	0.984	-
EE	0.009	3	2	0.398	-
AF*	0.007	0	4	-	-
BOVC1	0.007	2	2	0.705	-
CO*	0.007	0	4	-	-
CK*	0.007	0	4	-	-
BF	0.005	1	2	0.805	-
AOVC1*	0.005	0	3	-	-
AJ*	0.004	0	2	-	-
Ak	0.004	1	1	0.787	-
CMI-2*	0.004	0	2	-	-
EK	0.004	1	1	0.787	-
EO*	0.004	2	0	-	-
GG*	0.004	0	2	-	-
KO*	0.004	2	0	-	-
BJ*	0.002	0	1	-	-
DK*	0.002	1	0	-	-
DO*	0.002	0	1	-	-
EF*	0.002	0	1	-	-
EG*	0.002	0	1	-	-
EOVC1*	0.002	0	1	-	-
EMI-2*	0.002	0	1	-	-
GMI-2*	0.002	0	1	-	-
GO*	0.002	0	1	-	-
KK*	0.002	1	0	-	-
CMI-1*	0.002	0	1	-	-
FMI-2*	0.002	0	1	-	-
Paired Control Sampling					
AA	0.059	5	1	0.321	-
AB	0.225	14	9	-	-
AC	0.108	6	5	0.726	-
AD	0.059	2	4	0.24	-
AE	0.01	1	0	-	-
AK	0.01	1	0	-	-
AOVC1*	0.01	0	1	-	-
AMI-2	0.01	1	0	-	-
AO	0.01	1	0	-	-
BB	0.078	6	2	0.476	-
BC	0.137	2	12	0.011	0.107 (0.014–0.510)
BD	0.02	1	1	0.765	-
BE	0.059	4	2	0.795	-
BG	0.02	2	0	-	-
BK	0.01	1	0	-	-
BMI-2	0.01	1	0	-	-
BO	0.01	0	1	-	-
CC	0.029	1	2	0.382	-
CD	0.039	0	4	-	-
CK	0.01	0	1	-	-
CMI-1	0.01	0	1	-	-
DD	0.01	0	1	-	-
EC	0.01	0	1	-	-
ED	0.01	1	0	-	-
EE	0.029	1	2	0.382	-
MI-2 F	0.01	0	1	-	-

**Figure 2.**
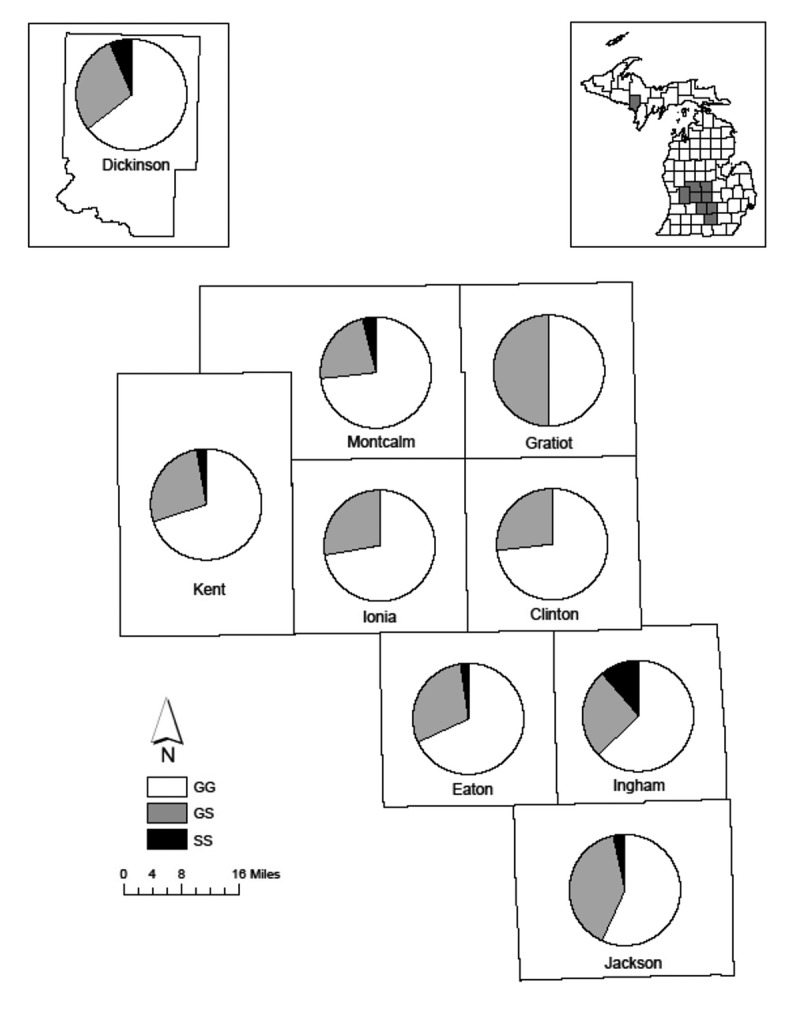
Proportion of *PRNP* amino acid 96 genotypes of white-tailed deer (*Odocoileus virginianus*) from 9 CWD-positive Michigan counties

Three genotypes at aa96 were observed; aa96GG was most common (*n* = 387) and was used as the reference in logistic regression. aa96GS was less likely to be found in deer infected with CWD (OR = 0.360 and 95% CI = 0.227–0.556, P < 0.001; Table 4; Figure 2); however, we did not detect a reduced likelihood of infection for homozygous individuals (aa96SS). Two genotypes at aa95 were observed, aa95QQ was most common (*n* = 552). No significant associations were seen for genotype at aa95 and CWD infection.

**Table 4.** *PRNP* genotype frequency (f) and count at codons 95 and 96 for chronic wasting disease positive (+) and non-detected (-) white-tailed deer (*Odocoileus virginianus*) from 9 CWD-positive Michigan counties

Codon	Genotype	f	(+)	(-)	P-val	Odds Ratio
95	QQ	0.972	184	368	-	-
	QH	0.028	1	15	0.052	0.133 (0.007–0.665)
96	GG	0.681	149	238	-	-
	GS	0.287	30	133	<0.001	0.360 (0.227–0.556)
	SS	0.032	6	12	0.66	-

Among the case-controlled samples, the presence of one C haplotype or one serine at aa96 was confirmed to be associated with reduced CWD infection by 0.191 (95% CI = 0.065–0.555, P < 0.01) and 0.182 (95% CI = 0.063–0.528, P < 0.01), respectively. As with the full dataset comparison, no evidence for protection was seen in homozygous CC or aa96SS individuals.

The ratio of haplotypes C and F, diplotype BC, and genotype aa96GS (associated with reduced susceptibility) relative to non-protective haplotypes, diplotypes and genotypes, respectively, were compared among the nine studied counties. Pairwise comparisons using Fisher’s exact tests failed to detect significant differences among counties in the distribution of protective genetic types after p-values were corrected for multiple comparisons.

## Discussion

This is the first examination of *PRNP* variation for a wild white-tailed deer population in Michigan. We established baseline frequencies of *PRNP* genotypes, haplotypes, and diplotypes in nine known CWD-positive counties. We found aa96GS, haplotypes C and F, and diplotype BC to be less frequent in CWD-positive deer, consistent with other studies [22, 24–27, 29–35]. The results of our analyses of paired samples controlling for potential confounding variables of age, sex, and harvest location further reinforce the finding of an association between haplotype C and the presence of a serine at aa96 with reduced vulnerability to CWD. While the C haplotype and aa96S were less frequent in CWD-positive deer, we did not find evidence to support a reduced vulnerability to CWD for homozygotes. Previous work has also failed to detect a reduced likelihood of infection among aa96SS individuals [22]. In the current study, this could be indicative of a biological process due to strain type, or an artefact of the low prevalence of aa96SS reducing our power to detect an effect. To account for strain-type differences, these results should be used to target aa96GG, aa96GS, and aa96SS CWD-positive individuals for inclusion in strain-type assessments. And with increased sampling over time we may produce a greater proportion of aa96SS individuals for evaluation.

Annual apparent CWD prevalence between 2015 and 2019 varied across the 9 positive counties with the highest prevalence of 1.95% seen in Kent county in 2019 (Table 5). We found no statistically significant differences in *PRNP* genotype or haplotype frequencies across these counties suggesting that, within the current known distribution of CWD in Michigan, infection vulnerability based on *PRNP* is relatively homogeneous. Given that deer in some counties in Michigan seem to have higher CWD prevalence than others, it will be of interest to monitor the potential selective impacts of CWD across these areas. While no *PRNP* types have been associated with complete resistance, the presence of aa96S has been associated with slower disease progression and longer survival post-infection [30, 31]. Longer survival may provide deer with aa96S a selective advantange leading to changes in *PRNP* frequencies in wild populations over time [27]. Our data present the current localized prevalence of G96S (28.7%) as similar to studies in white-tailed deer in Wyoming [38] (20%), but higher than those in Illinois [33] (13.8%), and northern Illinois and southern Wisconsin [22] (11%). Our characterization of *PRNP* frequencies, presumably relatively early in the disease’s occurrence in Michigan, provides a baseline for monitoring selective effects of CWD on *PRNP* frequencies and white-tailed deer population characteristics over time and should be used in disease modelling efforts to map risk and rate of spread.

**Table 5.** Apparent prevalence of chronic wasting disease in white-tailed deer (*Odocoileus virginianus*) from 9 CWD-positive Michigan counties. Number in parentheses corresponds to total number of animals tested for the year in the given county

County	2015	2016	2017	2018	2019	Total
Clinton	0.19% (1038)	0.07% (2716)	0 (2843)	0.06% (1737)	0 (908)	0.05% (9242)
Dickinson	-	0 (144)	0 (212)	0.14% (716)	0 (553)	0.06% (1625)
Eaton	0 (138)	0 (432)	0 (589)	0.13% (744)	0 (528)	0.04% (2431)
Gratiot	0 (28)	0 (49)	0 (144)	0.09% (1146)	0.29% (1045)	0.17% (2412)
Ingham	0.14% (2147)	0.09% (2110)	0 (2038)	0 (1599)	0 (824)	0.06% (8718)
Ionia	0 (18)	0 (300)	0 (899)	0.10% (1928)	0.21% (958)	0.10% (4103)
Jackson	0 (17)	0 (53)	0 (46)	0.13% (1546)	0.41% (1713)	0.27% (3375)
Kent	0 (3)	0 (21)	1.83% (546)	0.59% (1526)	1.95% (871)	1.21% (2967)
Montcalm	0 (16)	0 (48)	0.93% (3772)	1.12% (4009)	1.84% (1961)	1.18% (9806)
	0.15% (3405)	0.07% (5873)	0.41% (11,089)	0.41% (14,951)	0.69% (9361)	0.41% (44,679)

It is important to note some possible limitations to our study that point towards the need for future investigation. This assessment was a snapshot of polymorphisms restricted to a 625bp region from deer in a relatively small geographic area. Future work to monitor frequencies of haplotype C, aa96S, and any new informative polymorphisms outside of the 625bp region will help inform disease impact, possible selection within the population, and target regions for special management attention. Additional assessments of genetic connectivity among deer in the CWD-positive regions would also inform delineation of management areas.

Surveillance is currently being used as the leading indicator to inform CWD management in wild deer populations, and while beneficial, surveillance is costly, limited in scope, and is not in itself a management tool. As CWD detections continue to increase the areas under surveillance, the use of regionally specific data to allocate testing efforts and funding will be pivotal for success. Identification of *PRNP* polymorphisms associated with reduced vulnerability to CWD and their spatial distribution and prevalence may help managers design surveillance programmes to identify and prioritize areas for CWD management when partnered with movement data and anticipated deposition of prions onto the landscape over time.

## Materials and methods

### Sampling

Medial retropharyngeal lymph nodes were collected from white-tailed deer by Michigan Department of Natural Resource staff during routine disease surveillance between April 2015 and January 2020 from 9 CWD-positive Michigan counties. Sex, harvest location, and age, as assessed by tooth wear and replacement, were collected from all sampled deer. Samples were stored at −20°C or −80°C until prepared for DNA extraction.

Subsampling within each county for this study represented: 1) CWD-positive deer; 2) CWD non-detected deer; and 3) and additional CWD non-detected paired controls. Sampling aimed to obtain three individuals from unique sections (2.6 km^2^) in each township (93 km^2^) for CWD non-detected animals. To control for factors known to be associated with CWD infection probability, paired controls were identified for a subset of CWD-positive deer by matching a CWD-positive deer to a CWD non-detected deer of the same age, sex, and harvest location. Due to the already small sample size for paired controls, we were unable to control for background relatedness as done previously [42].

Samples were collected within a short period of time that led us to assume relatively similar exposure to CWD between paired case-controls and CWD-positive and CWD non-detected deer.

### CWD diagnosis

All animals were tested for CWD using a USDA approved enzyme-linked immunosorbent assay to detect PrP^CWD^ at either the Michigan (East Lansing, MI) or Wisconsin (Madison, WI) Veterinary Diagnostic Laboratory. Confirmation by immunohistochemistry was done by the diagnostic laboratories or by the National Veterinary Services Laboratory (Ames, IA). Sampling did not allow for the assessment of disease stage in different tissue types; however, the use of lymph tissue, where PrP^CWD^ deposition first occurs, reduced the chance that false negatives might impact these results [23].

### Prnp sequence analysis

Genomic DNA was isolated from lymph node tissue using Qiagen DNeasy Blood and Tissue Kits (Qiagen Inc., Valencia, CA) following manufacturer’s guidelines with a final elution volume of 200uL in Buffer AE.

The *PRNP* gene was amplified using a primer pair specific for the functional gene (223 5ʹ-acaccctctttattttgcag-3ʹ and 224 5ʹ-agaagataatgaaaacaggaag-3ʹ) [36]. PCR amplicons were purified using ExoSAP-IT (Applied Biosystems, Foster City, CA) and products were sequenced using the Big Dye Terminator system (Applied Biosystems, Foster City, CA). Sequence products were purified using ethanol/EDTA precipitation and resolved on an ABI 3500.

Sequences were visualized and edited in SEQUENCHER (Gene Codes Corporation, Ann Arbor, MI). Re-sequencing was done until regions of variability were confirmed three times. Haplotypes were generated from unphased sequences using DNA Sequence Polymorphism 5.10.01 (Rozas et al., Universitat de Barcelona). Markov chain Monte Carlo (MCMC) samples were taken from a minimum of 1,000 iterations, with a discarded burn-in of 100 iterations. Previously published haplotype sequences [22, 43] were uploaded from NCBI and a local BLAST was run to match phased sequences to published haplotypes.

Phased sequences were translated in SEQUENCHER to their amino acid composition for final reporting.

### Statistical analyses

We used logistic regression to identify associations between CWD status and haplotype, diplotype, and aa95 and aa96 genotypes. Chronic wasting disease status was a binomial variable with CWD-positive deer coded as 1 and non-detected deer coded as 0. Genetic data were treated as categorical variables. The most common genetic type was used as the reference type in each analysis. Genetic types significantly associated with CWD status were those with P-values ≤ 0.05. Odds ratios (ORs) and associated 95% confidence intervals were also calculated. Odds ratios with 95% confidence intervals that did not include one were considered significant. Genetic types with significant ORs less than one were interpreted as exhibiting reduced susceptibility to CWD.

To further explore associations between CWD status and genetic type while controlling for other factors that might affect CWD status (eg, age, sex, location), conditional logistic regression was used to identify associations between CWD status and genetic types for matched case-control pairs. The lesser number of available pairs (*n* = 51) limited the analyses we could conduct. Based on findings from the analyses described above as well as previous studies, we tested for associations between CWD status and presence of a C haplotype, CC genotype, and presence of at least one serine at aa96 using the clogit function in the survival package [44] in R [45] (version 3.6.1). We coded CWD status as described above. We did not assess haplotype F as only one available deer pair had a F haplotype. Significance was interpreted as described above.

We assessed differences in the frequency of presumably protective haplotypes, diplotypes, and genotypes among the 9 counties where CWD had been identified using Fisher’s exact tests.
